# Towards High Performance: Solution-Processed Perovskite Solar Cells with Cu-Doped CH_3_NH_3_PbI_3_

**DOI:** 10.3390/nano14020172

**Published:** 2024-01-12

**Authors:** Abdul Kareem Kalathil Soopy, Bhaskar Parida, S. Assa Aravindh, Asma O. Al Ghaithi, Naser Qamhieh, Noureddine Amrane, Maamar Benkraouda, Shengzhong (Frank) Liu, Adel Najar

**Affiliations:** 1 Department of Physics, College of Science, United Arab Emirates University, Al Ain 15551, United Arab Emirates; abdulkareemks@gmail.com (A.K.K.S.); bhaskar.parida@gmail.com (B.P.); 201050022@uaeu.ac.ae (A.O.A.G.); nqamhieh@uaeu.ac.ae (N.Q.); namrane@uaeu.ac.ae (N.A.); maamar@uaeu.ac.ae (M.B.); 2 Nano and Molecular Systems Research Unit (NANOMO), University of Oulu, Pentti Kaiteran Katu 1, 90570 Oulu, Finland; assa.sasikaladevi@oulu.fi; 3 Dalian National Laboratory for Clean Energy, Dalian Institute of Chemical Physics, Chinese Academy of Sciences, Dalian 116023, China; 4 Center of Materials Science and Optoelectronics Engineering, University of Chinese Academy of Sciences, Dalian 116023, China; 5 Key Laboratory of Applied Surface and Colloid Chemistry, Ministry of Education, Shaanxi Engineering Lab for Advanced Energy Technology, School of Materials Science and Engineering, Shaanxi Normal University, Xi’an 710119, China

**Keywords:** perovskite, doped perovskite, Cu^+^ ions, power conversion efficiency

## Abstract

Perovskite solar cells (PSCs) have demonstrated remarkable photovoltaic performance, positioning themselves as promising devices in the field. Theoretical calculations suggest that copper (Cu) can serve as an effective dopant, potentially occupying interstitial sites in the perovskite structure, thereby reducing the energy barrier and enhancing carrier extraction. Subsequent experimental investigations confirm that adding CuI as an additive to MAPbI_3_-based perovskite cells improves optoelectronic properties and overall device performance. Optimizing the amount of Cu (0.01 M) has been found to significantly enhance crystalline quality and grain size, leading to improved light absorption and suppressed carrier recombination. Consequently, the power conversion efficiency (PCE) of Cu-doped PSCs increased from 16.3% to 18.2%. However, excessive Cu doping (0.1 M) negatively impacts morphology, resulting in inferior optical properties and diminished device performance. Furthermore, Cu-doped PSCs exhibit higher stabilized power output (SPO) compared to pristine cells. This study underscores the substantial benefits of Cu doping for advancing the development of highly efficient PSCs.

## 1. Introduction

Halide perovskites have revolutionized the field of photovoltaics and related optoelectronics as a result of their unique optoelectronic properties [1,2,3,4]. These perovskites are multifunctional materials synthesized from inexpensive starting compounds that are abundant in nature [5]. Using the AMX_3_ formula, hybrid perovskites are described, in which A represents an organic cation, for example, methylammonium CH_3_NH_3_ (MA) and formamidinium NH_2_CH_3_NH_2_ (FA). M, on the other hand, is a divalent metal such as lead (Pb) or tin (Sn). It is important to note that X is a halide anion, like chlorine (Cl), bromine (Br), and iodine (I) [6,7]. They have a broad range of morphologies, distinctive photophysical properties, high carrier mobility, and long carrier diffusion length, all of which are extremely fascinating features that combine the admirable qualities of organic as well as inorganic materials [8,9]. Furthermore, they can be easily processed using several techniques, such as spin coating, dip coating, thermal evaporation, and chemical vapor deposition [10,11,12]. As a result of their unique characteristics and simple fabrication process, incredible research efforts have been made to enhance the power conversion efficiency (PCE) of the PSCs utilizing the chemical engineering process and implementing several device architectures [13,14,15,16,17,18]. Thus, the PCE of the PSCs has significantly improved from 3.8% to 25.8% in just over a decade [19,20].

In typical organic–inorganic perovskites, the chemical composition and the nature of the material are crucial for customizing the electronic properties, optical bandgaps, device performance, and stability [21]. Recent studies have demonstrated that the structural stability of the perovskite film is mostly controlled by the organic cation CH_3_NH_3_^+^ and is not directly influenced by structure bonding [22]. The outer orbitals of the divalent metal and halide, however, have the greatest impact on the electrical properties. The upper valence band is primarily generated by halogen *p* orbitals combined with Pb *s* orbitals, whereas the perovskite conduction band is primarily derived from the vacant Pb *p* orbitals. As a result of Pb often being fixed, X can adjust the band gap of the perovskite material in a wide range [21,22,23]. Thus, the complete or partial substitution of Pb or doping with homo- or hetero-valent cations can influence the perovskite material properties and photovoltaic performance, such as the band gap, the light absorption coefficient, and the charge carrier diffusion length [24].

Previous works on the effects of the partial substitution of the Pb^2+^ ions at the perovskite crystal lattice have shown that controlling the crystallization and the optoelectronic characteristics of perovskites is feasible via the incorporation or partial substitution of Pb with a monovalent cation such as Cu, Ag, K, and Na [25]. These ions were found to have reduced the trap-assisted non-radiative recombination of perovskite films, enhanced the crystallization, and increased the carrier lifetime. Additionally, doping Pb using divalent cations such as Sn [26], Zn [27], Sr [28], and Cd [29] has been shown to improve the crystal quality, enlargement of the grain size, tuning of optical band gaps, and enhancement in the carrier lifetime of the perovskite. Furthermore, doping the perovskite with a trivalent cation such as Bi [30] and Al [31] has shown a similar modification in the crystallization and the optoelectronic properties. There are only a few studies that investigated the inclusion of Cu^+^ ions, which have an ionic radius comparable to that of Pb^2+^, into perovskite precursor solutions [22,26]. However, there is no extensive study combining experimental data and density functional theory calculation to understand the position of Cu atoms in the Cu-doped perovskite and density of states calculation to understand the energy levels and their effect on electronic properties and device performance.

In this study, we used CuI as a dopant for the MAPbI_3_-based perovskite. Cu-doping into the perovskite significantly improved the uniformity, grain size, and crystal quality of the perovskite film, which substantially improved the light absorption and reduced the non-radiative recombination. We also employed a DFT calculation to determine the position of Cu atoms in the perovskite films. The DFT calculation revealed that Cu prefers to occupy the interstitial site and reduces the energy barrier by reducing the work function of the perovskite film, which significantly enhanced carrier extraction. As a result, the photovoltaic parameters of the Cu-doped PSCs increased with a PCE of 18.2% compared to the pristine solar cell (16.3%).

## 2. Materials and Methods

All materials used in this study were obtained commercially and used as received. Lead iodide (PbI_2_, 99.9985%), methyl ammonium iodide (MAI), N, N-dimethylformamide (DMF; 99%), Dimethyl sulfoxide (DMSO; 99.9%) ethylene glycol (99.5%, ethylenediamine (EDA, 99.0%,), Chlorobenzene (99.8%), and Nickel nitrate hexahydrate (Ni(NO_3_)_2_·6H_2_O) were bought from Sigma Aldrich, Saint Louis, MI, USA. Phenyl-C61-butyric acid methyl ester (PCBM, 99.5%) was purchased from Nano-C, Westwood, MA, USA.

*Precursors preparation-HTLs and ETLs*: Undoped and doped NiOx HTLs were synthesized using the solution processing according to the prior report [32]. Briefly, 0.291 g of Ni(NO_3_)_2_.6H_2_O was dissolved in ethylene glycol (1 mL) and ethylenediamine (72 µL) as an additive and stirred at room temperature (RT) overnight to produce the undoped NiOx. In order to produce undoped NiOx, 0.291 g of Ni(NO_3_)_2_.6H_2_O was dissolved in ethylene glycol (1 mL) with ethylenediamine (72 µL) as an additive and stirred at room temperature (RT) for an overnight period. PCBM (20 mg/mL) was dissolved in CB and stirred at RT overnight.

*Fabrication of perovskite solar cells*: Inverted planar pristine and Cu-doped PSCs were fabricated using a device structure of FTO/NiOx/MAPbI_3_ or Cu-doped MAPbI_3_/PCBM/Ag. Before drying in an oven, FTO substrates were cleaned sequentially for 10 min each with a detergent solution, DI water, acetone, and isopropanol (IPA). The substrates were cleaned, dried, and then given a 30-minute treatment with ozone (O_3_) plasma to improve their surface wettability. The NiOx HTL was prepared by spin-coating a precursor onto the FTO substrates at 4000 rpm for 90 s and annealing them for 1 h at 300 °C in an ambient air atmosphere. After cooling to room temperature, the substrates were transferred to a glove box filled with nitrogen where the air and water content was <1 ppm, and then the MAPbI_3_ perovskite layer was coated. The MAPbI_3_ perovskite precursor solution was synthesized by dissolving MAI (1.1 M) and PbI_2_ (1.1 M) in a mixed solvent of DMF: DMSO (0.7:0.3 mL) and stirred for 4 h at RT. Similarly, Cu-doped MAPbI_3_ solutions were prepared by adding 0.01, 0.1, 0.03, 0.08, and 0.1 M of CuI into the perovskite solution. A PVDF (0.45 µM, Whatman) filter was used to filter the perovskite precursor solution before it was spin-coated onto the HTL layer at 3800 rpm for 20 s. Once the spinning was ready to stop, 300 µL of CB as an antisolvent was dropped onto the perovskite film. The antisolvent-treated MAPbI_3_ samples were then spin-coated again at 5000 rpm for 20 s, followed by an immediate heat treatment at 100 °C for 10 min. The MAPbI_3_ perovskite film was then spin-coated with PCBM solution for 30 s at 3000 rpm. After setting a shadow mask to define an effective cell area of 0.04 cm^2^, 120 nm of Ag electrodes were finally deposited on top of the devices using a thermal evaporator at 2.2 × 10^−6^ torr.

*Film and device characterizations*: X-ray diffraction (XRD; R&D-100; Rigaku SmartLab, Akishima-shi, Tokyo, Japan) was utilized to analyze the structural characteristics of the perovskite films. We examined the surface and cross-sectional morphologies of the synthesized ANO and perovskite films using a field-emission scanning electron microscope (FE-SEM; SIGMA, Carl Zeiss, Oberkochen, Germany). Planar perovskite films’ absorption spectra were evaluated using UV-visible (UV-vis) spectrophotometry (UV-2700; Shimadzu, Kyoto, Japan). A spectrofluorometer (FP-8600, Jasco, Easton, MD, USA) was employed to conduct steady-state PL measurements of the fabricated perovskite films at a laser excitation wavelength of 530 nm. Using a fluorescence spectrometer (FlouTime 300, PicoQuant, Berlin, Germany) with a laser excitation wavelength of 398.1 nm, time-resolved photoluminescence (TR-PL) studies of the fabricated MAPbI_3_-perovskite films on the ANO-based HTL films were carried out. Using a solar simulator (PEC-L01, Peccell Technologies, Yokohama, Japan), the current–voltage (J–V) curves and the steady-state photocurrent of the fabricated PVSCs were measured under standard AM 1.5 illumination (100 mW/cm^2^) in ambient air conditions. To detect responses as a function of the spectral wavelengths, the external quantum efficiency (EQE) spectrum was evaluated using a monochromator (DongWoo Optron, MonoRa500i, Taipei, Taiwan), a power source (Abet Technologies 150 W Xenon lamp, Milford, CT, USA), and a CompactStat (Ivium Technologies; Eindhoven, The Netherlands).

*Computational Methods*: Density functional theory (DFT) simulations were performed using the plane wave pseudopotential code, Vienna Ab initio Simulation Package (VASP), to validate some of our experimental results [33]. A kinetic energy cutoff of 650 eV and Gaussian smearing were used to expand the plane waves included in the basis set. The exchange and correlation interactions were described by the generalized gradient approximation (GGA) in the PAW-PBE approach [34,35]. The pseudopotentials used were of the Projected Augmented Wave formalism (PAW). The atomic positions were relaxed with energy and force tolerances of 10–6 eV and 0.001 eV/Å, respectively. The supercell approach is used to model a (2 × 2 × 1) supercell of MAPbI_3_ containing 48 atoms and a Monkhorst Pack k grid of 4 × 4 × 8 was used for Brillouin zone integration. To account for the halide atom, we included the Hubbard parameter with the values of U = 8 eV and J = 0 eV [36].

## 3. Results and Discussion

**Figure 1:**
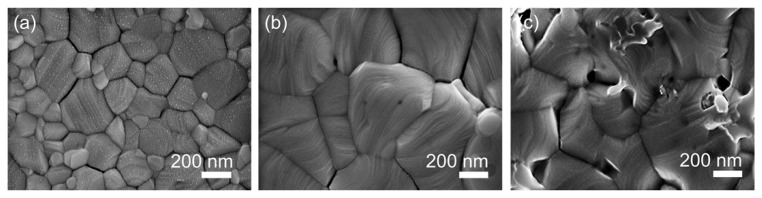
Top-view SEM images of (**a**) pristine, (**b**) 0.01 M, and (**c**) 0.1 M Cu-doped MAPbI_3_ films deposited on the FTO/NiOx layer.

To examine the surface morphology, films of pristine and Cu-doped (0.01 M and 0.1 M) MAPbI_3_ were deposited on FTO/NiOx layers and analyzed using FE-SEM. The top-view SEM image of the pristine perovskite film, as depicted in Figure 1a, reveals small-sized grains. In contrast, the perovskite film doped with 0.01 M Cu^+^, illustrated in Figure 1b, displays a film with larger grain sizes ranging from a few hundred nanometers to over 1 μm, featuring a homogeneous, pinhole-free surface. However, Figure 1c illustrates the perovskite film doped with 0.1 M Cu^+^ ions, revealing a poorly covered surface with significant roughness. These findings suggest that a modest amount of Cu^+^ ion doping in the perovskite precursor solution can enlarge grain size, mitigate defects arising from grain boundaries, and enhance charge carrier lifetime [37]. The average grain size for the pristine sample was calculated as 212 nm, while the sample doped with 0.01 M Cu^+^ exhibited an average grain size of 369 nm. The particle size distribution histogram, derived from SEM images, is presented in Figure S1 (Supporting Information). However, perovskite films doped with a higher concentration of Cu^+^ ions can deteriorate the crystal structure, trapping charge carriers and diminishing the power conversion efficiency (PCE) of the solar cells.

**Figure 2:**
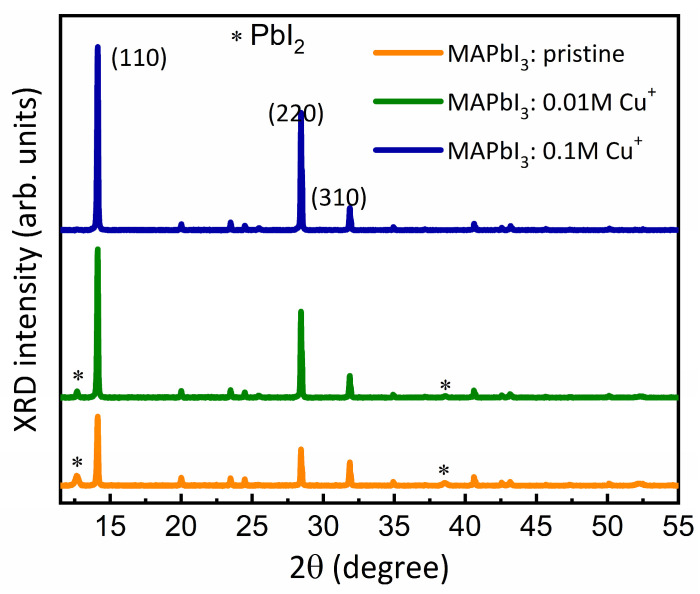
XRD patterns of pristine, 0.01 M, and 0.1 M Cu-doped perovskite films. The * corresponded to lead iodide (PbI_2_) residue, resulting from an incomplete reaction between the perovskite precursor solution.

To investigate the influence of Cu doping on the crystallinity of MAPbI_3_ perovskite, the crystal structure was examined using an X-ray diffraction system. In Figure 2, the XRD patterns of pristine and Cu-doped MAPbI_3_ films display diffraction peaks at 14.2°, 28.5°, and 31.9°, corresponding to the (110), (220), and (310) crystal planes of the tetragonal phase of perovskite films. Remarkably, Cu-doped MAPbI_3_ samples show a significant increase in intensity for these peaks, aligning with the larger grains observed in the top-view SEM images. Interestingly, with the presence of 0.01 M Cu^+^, the diffraction peaks at 2θ = 12.6° and 2θ = 38.6°, corresponding to unconverted PbI_2_, (*) are reduced and eliminated. This suggests that low Cu^2+^ ion doping substitutes Pb^2+^ due to their small difference in ionic radii (Cu^2+^ = 73, Pb^2+^ = 119), enhancing the crystallinity of the perovskite film, as observed in the top-view SEM image (Figure 1b). However, beyond an optimal Cu^+^ ion addition level, the crystallization of MAPbI_3_ is substantially disrupted, leading to a higher impurity phase and increased defects. As doping increases, cation vacancies are formed, directly affecting lattice parameters and resulting in amorphous PbI_2_, evidenced by XRD peaks at 2θ = 12.6° and 38.6° [6,38,39] Notably, with no apparent shift in diffraction peak positions, the intensities of the prominent perovskite (110) and (220) peaks increase significantly with higher concentrations of Cu^+^ doping. This implies that the introduction of Cu^+^ ions aligns the crystal planes more effectively [39].

**Figure 3:**
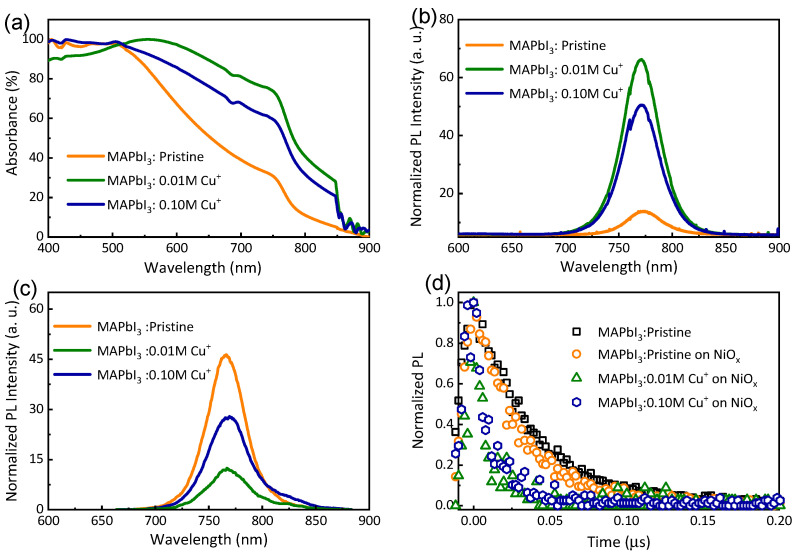
(**a**) UV-Vis absorption spectra, (**b**,**c**) PL spectra of pristine and Cu-doped MAPbI_3_ films deposited on glass and NiOx/FTO substrates, respectively. (**d**) Time-resolved PL decay spectra of pristine MAPbI_3_ film deposited on glass, and NiOx/FTO, 0.01 M, and 0.1 M of Cu-doped (blue dots) perovskite films deposited on NiOx/FTO.

The UV-Vis absorption spectra of pristine and Cu-doped (0.01 M and 0.1 M Cu) perovskite films are illustrated in Figure 3a. The optical absorption spectrum of the pristine perovskite film exhibits a broad absorption band covering the entire visible spectral range. Consequently, Cu-doped perovskite films display a significant enhancement in absorbance compared to the pristine film, suggesting that Cu doping has improved the quality of the perovskite film, aligning with the XRD and FE-SEM results.

The bandgap energy (Eg) of these samples is calculated using the Tauc plot, extrapolating the linear part of the plot to the x-axis, as presented in Figure S2 (Supporting Information). The pristine sample shows a bandgap energy of 1.70 eV, while the sample doped with 0.01 M Cu^+^ exhibits a lower bandgap energy of 1.33 eV. These results indicate that a lower bandgap energy corresponds to better absorption. Notably, perovskite films doped with 0.01 M Cu demonstrate the highest absorbance, whereas 0.1 M Cu leads to a decrease in absorption compared to the 0.01 M Cu-doped film. This is attributed to the 0.01 M Cu-doped perovskite film having uniform and larger grains compared to the film doped with 0.1 M Cu^+^ ions. Thus, these findings underscore that Cu^+^ doping can significantly enhance the light absorption capacity of the perovskite film, offering potential benefits for improving the power conversion efficiency (PCE) of corresponding perovskite solar cells (PSCs).

Figure 3b displays the photoluminescence (PL) spectra of pristine and Cu-doped MAPbI_3_ perovskite films deposited on a glass substrate. The MAPbI_3_ perovskite film doped with a small amount (0.01 M) of Cu^+^ ions exhibits the highest PL intensity compared to the 0.1 M Cu-doped and pristine films. This relatively high PL intensity indicates that 0.01 M Cu doping is the optimal condition, effectively reducing nonradiative recombination-related traps or defects. Conversely, pristine perovskite films show very low PL intensity, suggesting the presence of high nonradiative recombination centers. Excessive doping, higher than the pristine, results in a considerable decrease in PL intensity due to the development of more defect states. To further understand the carrier extraction properties, PL measurements of the perovskite films deposited on NiOx/FTO substrates were conducted, as illustrated in Figure 3c. Cu-doped perovskite films exhibit significant PL quenching compared to pristine films. The PL intensity reaches a minimum for the 0.01 M Cu-doped perovskite film, indicating effective extraction of photoexcited charge carriers by the substrate. Figure 3d presents the normalized time-resolved PL (TR-PL) kinetics of pristine and Cu-doped perovskite films. The pristine MAPbI_3_ perovskite deposited on the glass substrate exhibits an extended average lifetime, with a slight quenching when the hole transport layer (HTL) is introduced. In contrast, the lifetime of Cu-doped perovskite films deposited on NiOx is reduced to 3–4 ns, aligning with the PL spectra (Figure 3c). These findings demonstrate that Cu doping significantly reduces radiative recombination and enhances hole extraction [40].

**Figure 4:**
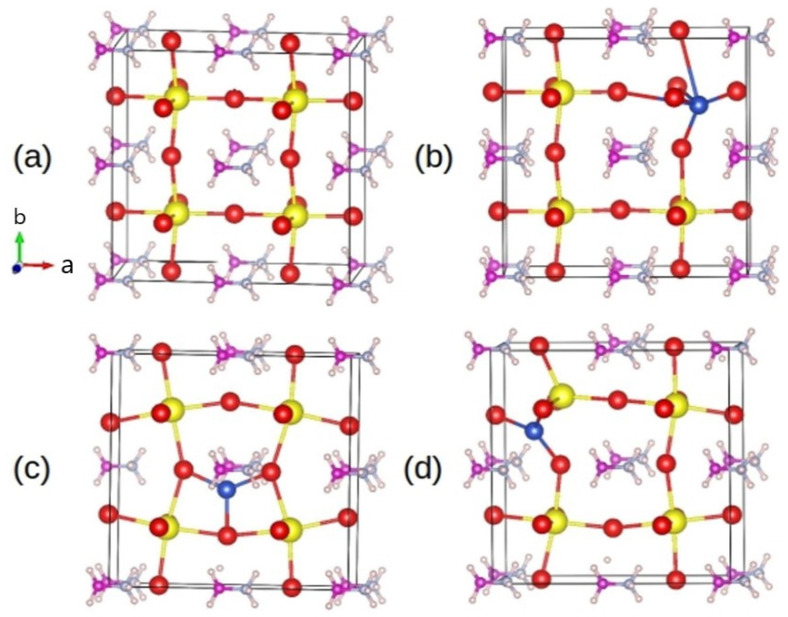
The relaxed geometries of the (2 × 2 × 1) MAPbI_3_ supercell. (**a**) Shows the relaxed geometry of the pristine supercell. (**b**) Cu substituted to Pb atom (**c**,**d**) represents Cu in two different interstitial positions. The color code of the atoms is pink: C, light blue: N, dark blue: Cu, red: I, yellow: Pb, and cream: H.

To scrutinize the role of Cu^+^ ions in the MAPbI_3_ perovskite and assess their impact on electronic behavior, density functional theory (DFT) calculations were performed. A (2 × 2 × 1) supercell of pristine MAPbI_3_ was chosen for optimization, yielding relaxed lattice parameters of a = 12.86 Å and c = 6.48 Å. One Cu atom was doped into the optimized supercell by substituting for the Pb atom, considering two interstitial positions, as presented in Figure 4. The formation energy and optimized Cu-I bond lengths are detailed in Table 1.

The formation energy is calculated by using the equations below,

*E*^*f*^ = *E*_*total*_ − *E*_*pure*_ + *μ*Pb − *μ*Cu (for Cu substitutional), (1)

*E*^*f*^ = *E*_*total*_ − *E*_*pure*_ − *μ*Cu (for Cu interstitial), (2)

Here, *E*_*total*_ denotes the total energy of the MAPbI_3_ supercell containing the Cu and *E*_*pure*_ is the total energy of pristine MAPbI_3_. *μ* represents the chemical potential of the respective element. From these equations, a negative formation energy indicates favorable adsorption and vice versa. It can be seen that the interstitial positions have lower formation energy compared to the substitutional and most stable configuration after relaxation, corresponding to Figure 4d, indicating that interstitial is the favored configuration. We see that when the Cu atom is placed in an interstitial configuration, the Cu-I horizontal bond lengths elongate to adjust the forces.

**Figure 5:**
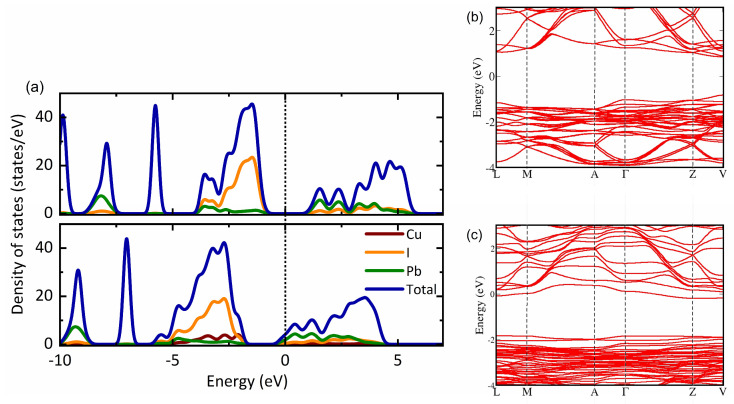
(**a**) The density of states (DOS) of MAPbI_3_ with and without Cu doping. The DOS is calculated for the most stable interstitial configuration. The band structure of (**b**) pristine MAPbI_3_ and (**c**) Cu-doped MAPbI_3_.

Additionally, an analysis of the electronic structure is conducted for the stable Cu configurations, as depicted in Figure 5. In Figure 5a, the total and atom-resolved density of states (DOS) for both the pristine and Cu-doped MAPbI_3_ are presented. The primary contribution to the DOS is observed from the halide atom. With the addition of Cu, there is a noticeable shift of the valence band maximum to lower energy regions. This shift aligns with the optical absorption measurements illustrated in Figure 3a, confirming that Cu serves as an acceptor impurity in this material. The band structure, depicted in Figure 5b,c, further emphasizes the evident shift of the valence band maximum to the lower energy region.

Furthermore, we conducted calculations to determine the work function for both MAPbI_3_ and Cu-MAPbI_3_, aiming to assess the impact of Cu addition. The work function (WF) is defined as WF = ϕ − EF, where ϕ represents the vacuum potential energy and EF is the Fermi energy. The calculated WF value for pristine MAPbI_3_ is 4.77 eV. In the case of Cu-MAPbI_3_, the calculated WF is 3.85 eV, which is lower than the experimentally measured value of 4.4 eV. However, it is noteworthy that the consistent finding of a reduced WF upon Cu^+^ doping of MAPbI_3_ is observed both experimentally and through DFT calculations. This reduction in WF for Cu-MAPbI_3_ implies a decrease in the energy barrier for the reaction with the addition of Cu.

**Figure 6:**
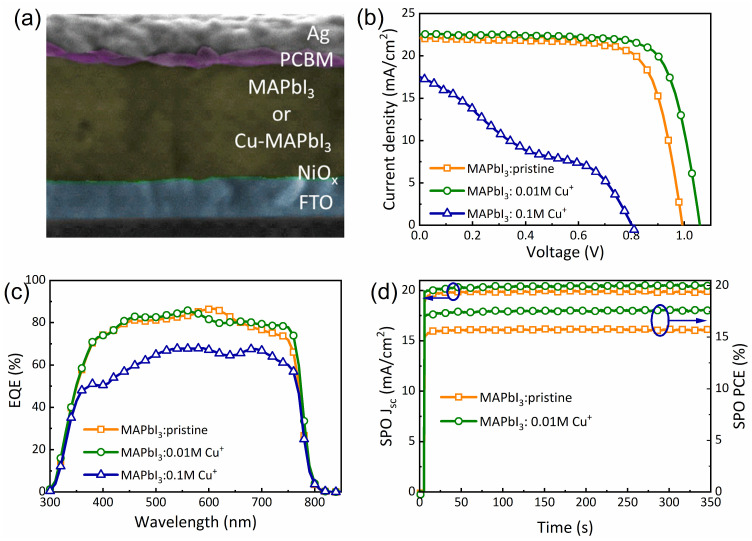
(**a**) SEM cross-sectional image of the inverted planar PSC showing device architecture of FTO/NiOx/MAPbI_3_ or Cu-MAPbI_3_/PCBM/Ag. (**b**) J–V and (**c**) EQE spectra of the pristine and 0.01 M and 0.1 M of Cu-doped PSCs. (**d**) Stabilized current density and PCE of the pristine and Cu-doped PSCs.

In order to investigate the impact of Cu doping on the performance of the PSCs, we embedded the Cu-MAPbI_3_ into solar cells sandwiched between NiOx as HTL and PCBM as ETL layers. Figure 6a shows the cross-sectional SEM image of the PSC with the device structure of FTO/NiOx/MAPbI_3_ or Cu-MAPbI_3_/PCBM/Ag. We have to highlight that 20 devices were tested for each concentration and for the reference cell. Figure 6b illustrates the current density–voltage (J–V) curves of both pristine and Cu-doped perovskite solar cells (PSCs), with corresponding photovoltaic parameters summarized in Table 2. The pristine PSC achieved an efficiency of 16.3%, accompanied by Jsc, Voc, and FF values of 22.1 mA/cm^2^, 0.99 V, and 74%, respectively. Notably, PSCs doped with 0.01 M Cu^+^ exhibited a significant improvement in PCE to 18.2% ± 0.51, with increased Jsc, Voc, and FF values reaching 22.5 mA/cm^2^, 1.06 V, and 76%, respectively. The enhancement of Voc and FF following Cu^+^ doping in a perovskite solar cell are critical parameters indicative of the potential difference between the cell’s electrodes in the absence of current flow and how well a solar cell can convert sunlight into electrical power, respectively. The observed increase in Voc and FF signifies an improvement in the cell’s capacity to generate higher voltage, likely stemming from advancements in improved crystal quality and grain growth, diminished defects, improved charge carrier dynamics, and enhanced interface properties [41,42]. This augmented Voc and FF contribute positively to the overall efficiency of the solar cell, particularly up to an optimal Cu^+^ doping concentration of 0.01%. However, for higher Cu^+^ doping concentrations (0.08% and 0.1%), a decline in Voc and FF values is evident. This reduction may suggest challenges such as increased recombination losses, perovskite degradation, or interface issues, leading to an overall decrease in efficiency and performance.

Furthermore, the PCE for PSCs doped with 0.005 and 0.03 M are 17.9% ± 0.57 and 18.1% ± 0.48, respectively. We can conclude that considering the margin of error, the PCE is approximately the same for the three Cu-doped concentrations. However, with a further increase in Cu^+^ concentration to 0.08 and 0.1 M, all photovoltaic parameters dramatically decreased, leading to a PCE drop to 4.6% ± 1.22 and 4.4% ± 1.15, respectively. This decline in performance is attributed to trap-assisted recombination caused by excess Cu acting as recombination centers. Additionally, the rough surface in the case of 0.1 M doped PSCs may result in inferior contact between the transport layers, negatively affecting charge collection efficiency. It is worth noting that the PCE of our Cu-doped PSCs surpasses that reported in previous studies [7,43,44,45,46].

Figure 6c presents the external quantum efficiency (EQE) spectra of both pristine and Cu-doped PSCs. Across the visible to near-infrared region (380–750 nm), all devices exhibit a broad range of EQE characteristics. The PSC doped with 0.01 M Cu displays relatively higher EQE compared to the pristine, owing to its improved photo-carrier extraction properties. Conversely, the PSC doped with 0.10 M Cu^+^ exhibits the lowest EQE values, attributed to the generation of defect centers through excess Cu^+^ doping, diminishing the carrier extraction properties. Stabilized current densities of our pristine and Cu-doped PSCs are depicted in Figure 6d, with biases maintained at 0.81 V and 0.88 V, respectively, close to the maximum power point under AM 1.5 illumination. Stable photocurrents of 19.7 and 20.1 mA/cm^2^ were achieved, corresponding to stabilized PCEs of 16.0% and 17.6% for the undoped and 0.01 M Cu-doped PSCs, respectively. These findings suggest that Cu doping in perovskite films significantly enhances the PCE stability of PSCs.

**Figure 7:**
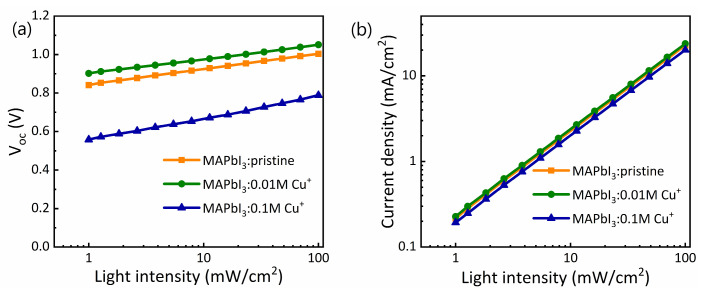
Light intensity-dependent (**a**) Voc and (**b**) Jsc of the pristine and Cu-doped PSCs.

To investigate the charge transportation and carrier recombination mechanism, we measured the light intensity-dependent V_oc_ and J_sc_ characteristics of the pristine and Cu-doped PSCs, as shown in Figure 7a and Figure 7b, respectively. We estimated the ideality factor (n) of our pristine and Cu-doped PSCs using the light intensity-dependent semilogarithmic plot of V_oc_, as shown in Figure 7a, and matched with a straight line using the following expression

(3) $$V_{OC}=\frac{\eta kT}{q}\ln\left(I\right)+constant$$

where *k* is the Boltzmann constant, *T* is temperature, *q* is electric charge and *I* is light intensity [47]. The proportionality factor (*ηkT*/*q*) can be determined based on the slope of this function. As a result, *η* = 1 indicates dominant bimolecular recombination (such as Langevin), while *η* = 2 indicates monomolecular, trap-assisted recombination (such as Shockley-Read-Hall (SRH)) [48]. Moreover, additional traps may emerge either within the perovskite layer or at the interface with the transport layer. The Cu-doped (0.01 M) device exhibits a relatively higher slope compared to the other devices, leading to a slightly higher Voc. By assessing the slopes, we estimated the ideality factor of the pristine, 0.01 M, and 0.1 M Cu-doped perovskite solar cells (PSCs) to be 1.35, 1.21, and 1.79, respectively. The Cu-doped (0.01 M) device demonstrates a reduced ideality factor, indicative of inhibited trap-assisted Shockley–Read–Hall recombination at the perovskite–hole transport layer (HTL) interface, attributed to improved charge extraction and reduced hole accumulation near the interface [49]. Conversely, the ideality factor increases with the introduction of an excess of Cu^+^ ions (0.1 M) to the perovskite, signifying an increase in trap-assisted recombination that adversely affects device performance. It is well-known that the short-circuit current density (Jsc) has a power-law dependence on light intensity Plight, expressed as Jsc α (Plight)^α^, where α represents the power-law exponent. Figure 7b depicts the light-dependent Jsc for our pristine and Cu-doped devices. When α = 0.75, the device operates in a space-charge-limited manner, and α close to 1 indicates a negligible space-charge limit [50]. We obtained α values of 1.06, 1.04, and 1.07 for the pristine, 0.01 M, and 0.01 M Cu-doped PSCs, respectively, in good agreement with a previous report [51]. All devices exhibit an α value close to 1, indicating effective inhibition of bimolecular recombination at the device interface. This suggests efficient elimination of charge carriers before recombination under short-circuit conditions [52,53,54]. Furthermore, it implies adequate electron and hole mobility with no charge transport barrier in solar cells, consistent with the high photo response observed in external quantum efficiency (EQE) [54].

## 4. Conclusions

In conclusion, we have successfully introduced Cu doping into MAPbI_3_-based perovskites and thoroughly investigated its impact on the structural, optical, electrical, and device performance of perovskite solar cells (PSCs). Our findings indicate that a judicious amount of Cu doping significantly enhances grain size and optical absorbance while reducing trap-assisted recombination in the perovskite films. However, excessive doping results in suboptimal device performance due to inferior grain size, which exacerbates trap formation in the absorber layer and compromises interfacial contact. DFT calculations further revealed that the addition of Cu shifts the valence band maximum to lower energy regions, reducing the energy barrier of the perovskite layer and facilitating carrier extraction. Consequently, the PCE improved efficiently from 16.3% (pristine) to 18.2% with 0.01 M of Cu doping. Thus, our study underscores the highly beneficial role of Cu doping in enhancing the quality of perovskite films and the PCE of PSCs, indicating its potential for the development of highly efficient PSCs on an industrial scale.

## Figures and Tables

**Table 1:** The formation energy and average Cu-I bond length of Cu-doped MAPbI_3_.

Doping Configuration	Formation Energy (eV)	Cu-I (Horizontal) (Å)	Cu-I (Vertical) (Å)
MAPbI_3_-Cu_*sub*_	1.63	2.49	2.63
MAPbI_3_-Cu_*int-1*_	−2.50	2.60	2.53
MAPbI_3_-Cu_*int-2*_	−2.48	2.55	2.54

**Table 2:** Photovoltaic performance of pristine and Cu-doped PSCs.

Solar Cells	J_sc_ (mA/cm^2^)	V_oc_ (V)	FF (%)	PCE (%)
Pure MAPbI_3_	−22.1 ± 0.55	0.99 ± 0.015	74.0 ± 2.11	16.3 ± 0.59
MAPbI_3_: 0.01 M of Cu^+^	−22.5 ± 0.87	1.06 ± 0.010	76.0 ± 1.73	18.2 ± 0.51
MAPbI_3_: 0.10 M of Cu^+^	−17.5 ± 0.23	0.79 ± 0.021	32.0 ± 2.56	4.4 ± 1.15

## Data Availability

Data are contained within the article.

## Supplementary Materials

The following supporting information can be downloaded at: https://www.mdpi.com/article/10.3390/nano14020172/s1, Figure S1: The histogram distribution of average grains of pristine perovskite and 0.01 M and 0.1 M Cu doped samples; Figure S2: Band gap energy (Eg) determination from the Tauc plot.
