# Supplementary material for: Towards High Performance: Solution-Processed Perovskite Solar Cells with Cu-Doped CH3NH3PbI3
Source: Nanomaterials (Basel). 2024 Jan 12;14(2):172. doi: 10.3390/nano14020172 (PMC10821043; doi:10.3390/nano14020172)
Supplement: Supplementary file 1 [file nanomaterials-14-00172-s001.zip › nanomaterials-2803650-supplementary.pdf]

Supporting Information

Towards High-Performance: Solution-Processed Perovskite Solar Cells with Cu-Doped $CH_3NH_3PbI_3$

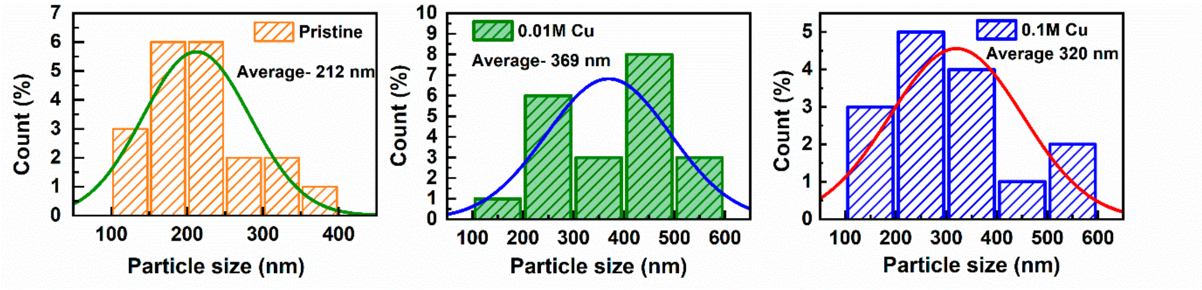

Figure S1. The histogram distribution of average grains of pristine perovskite and 0.01M and 0.1M Cu doped samples

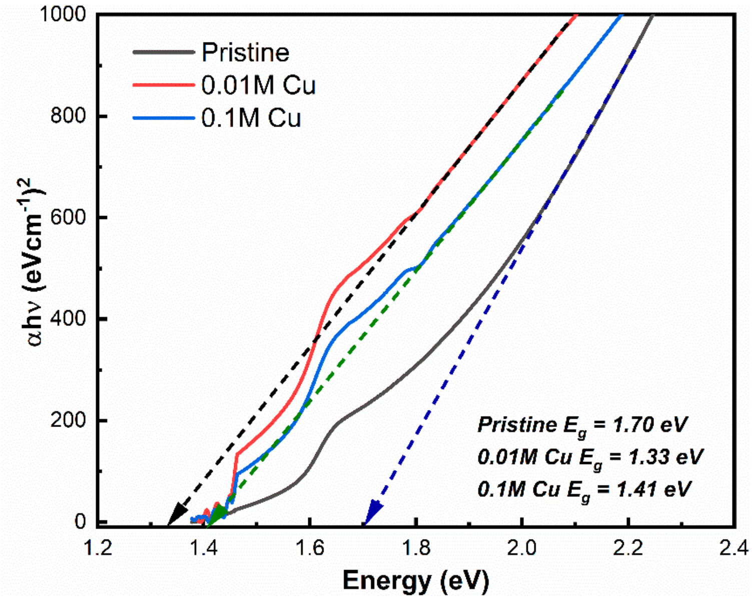

Figure S2. Band gap energy (Eg) determination from the Tauc plot.
